# Depressive symptoms mediate the association between frailty and motoric cognitive risk syndrome in Chinese adults: Evidence from CHARLS 2011–2013

**DOI:** 10.1097/MD.0000000000047166

**Published:** 2026-01-16

**Authors:** Jing Li, Yi-Xiao Li

**Affiliations:** a Department of Neurosurgery, Taikang Ningbo Hospital, Ningbo, China; b Department of Neurology, Taikang Ningbo Hospital, Ningbo, China.

**Keywords:** CHARLS, depressive symptom, frailty index, motoric cognitive risk syndrome

## Abstract

This study aimed to investigate the longitudinal association between the frailty index (FI) and motoric cognitive risk (MCR) syndrome among middle-aged and older adults, using nationally representative data from the China Health and Retirement Longitudinal Study. Data from the 2011 to 2013 China Health and Retirement Longitudinal Study waves were analyzed. A total of 3091 middle-aged and older adults aged ≥45 years were included in the study. Participants were categorized into the MCR and non-MCR groups. Multivariate logistic regression, restricted cubic spline regression, and subgroup analyses were performed to examine the association between FI and MCR. A mediation analysis assessed the role of depressive symptoms in this relationship. A significant association was observed between FI and MCR. Participants with an MCR exhibited higher FI levels than those without an MCR (*P* < .001). A positive linear relationship was identified between FI and MCR risk (*P* < .001). This association remained stable across all subgroups examined (*P* for nonlinearity = .924). Furthermore, depressive symptoms mediated the relationship between frailty and MCR, accounting for 32.1% of the total effect (*P* < .05). Our findings suggest a positive linear association between frailty and MCR prevalence in middle-aged and older Chinese adults, potentially mediated in part by depression. Incorporating frailty and depression assessments into geriatric evaluations may facilitate earlier identification and targeted interventions for individuals at an increased risk of MCR in aging populations.

## 1. Introduction

Motoric cognitive risk (MCR) syndrome, a pivotal pre-dementia condition, is defined by subjective cognitive complaints co-occurring with slow gait in older adults without dementia or mobility disability.^[1]^ With a global prevalence ranging from 2% to 18%,^[2]^ MCR poses a significant health burden due to its robust associations with adverse outcomes including falls, disability, dementia, and increased mortality.^[3-5]^ Studies indicate that MCR confers a significantly higher risk of cognitive decline and dementia than either slow gait speed or subjective memory complaints alone, highlighting the imperative for early identification and intervention.^[6]^ Given these serious consequences and the current lack of effective preventive treatments, identifying modifiable risk factors for MCR becomes an urgent public health priority.

One promising yet incompletely understood modifiable factor is frailty. This clinically significant geriatric syndrome is characterized by multisystem physiological decline, heightening vulnerability to stressors.^[7]^ Its prevalence increases with age, with a global pooled prevalence of approximately 23.9% among older adults.^[8]^ Frailty is strongly associated with multiple adverse health outcomes including physical disability,^[9]^ Alzheimer’s disease,^[10]^ and mortality.^[11]^ While previous studies have demonstrated an association between frailty and an increased risk of incident MCR in older individuals,^[12,13]^ several critical limitations persist. First, existing research has predominantly focused on either adults aged ≥60 years,^[12]^ or ≥65 years,^[14]^ or utilized multiethnic cohorts.^[13]^ Moreover, previous studies employed varied frailty metrics (e.g., clinical frailty scale^[12]^), making cross-study comparisons of the frailty-MCR association strength challenging. These inconsistencies underscore the need to standardize quantification of frailty-MCR association, particularly in underrepresented middle-aged and older Chinese populations.

Beyond mechanistic hypotheses involving inflammation,^[15]^ depression represents another significant pathway requiring exploration. Recent studies have shown that depression is a risk factor for MCR development in older adults.^[16,17]^ Moreover, genetic evidence supports a bidirectional causal association between frailty and depression,^[18]^ suggesting a potential novel pathway linking frailty to MCR. This study aimed to investigate the longitudinal association between frailty and MCR in middle-aged and older Chinese adults using data from the China Health and Retirement Longitudinal Study (CHARLS). In addition, we investigated the potential mechanistic pathways linking frailty to MCR risk through a mediation analysis of depressive symptoms.

## 2. Materials and methods

### 2.1. Study population

CHARLS is a nationally representative cohort comprising community-dwelling adults aged 45 years and older in China and employs a multistage stratified probability-proportional-to-size sampling strategy. Between June 2011 and March 2012, 17,705 participants from 10,257 households across 450 villages within 150 county-level units spanning 28 provinces were enrolled. Trained interviewers conducted face-to-face interviews using standardized questionnaires to collect comprehensive data on sociodemographic characteristics, lifestyle behaviors, and health metrics. To date, CHARLS has completed 4 waves of data collection: the national baseline survey (wave 1, 2011), first follow-up survey (wave 2, 2013), second follow-up survey (wave 3, 2015), and third follow-up survey (wave 4, 2018). All data are publicly accessible via the CHARLS website (http://charls.pku.edu.cn/en). The CHARLS survey project was approved by the Biomedical Ethics Committee of Peking University, and written informed consent was obtained from all the participants. This study strictly adhered to the Strengthening the Reporting of Observational Studies in Epidemiology (STROBE) guidelines.^[19]^

**Figure 1.**
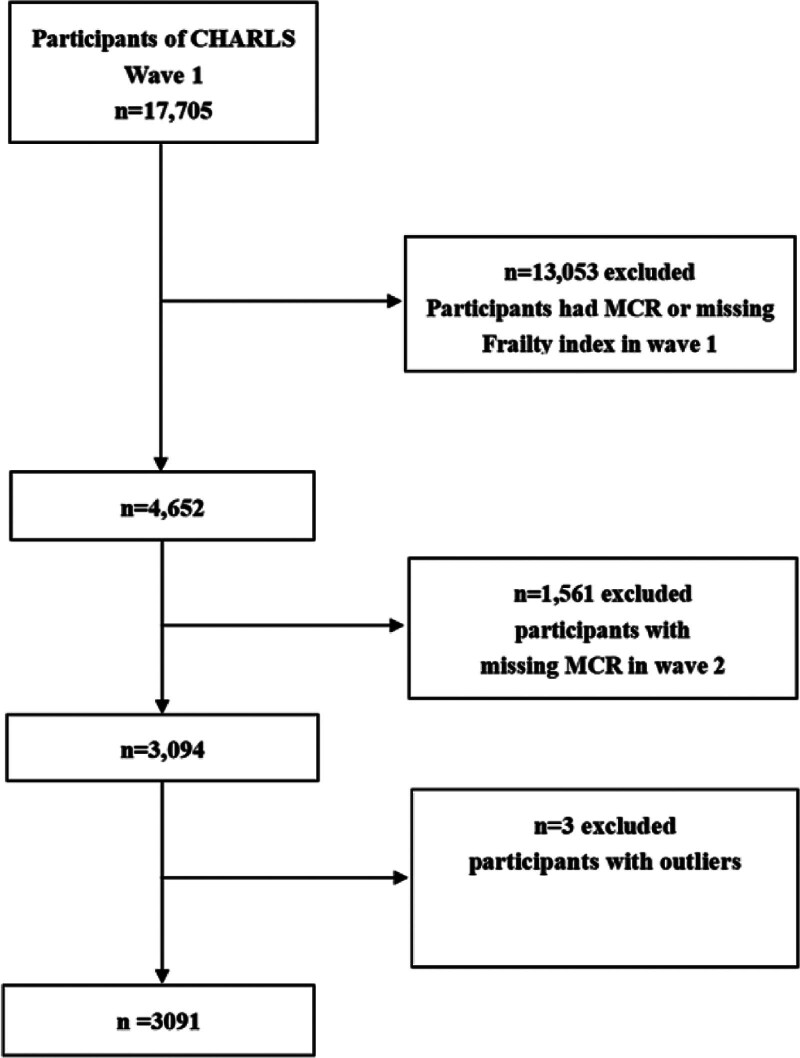
The flowchart of participants selection. CHARLS = China Health and Retirement Longitudinal Study, MCR = motoric cognitive risk.

This study used data from 2 waves collected in 2011 (wave 1) and 2013 (wave 2), respectively. For the current analysis, we restricted the participants to those aged 45 years or above. The sample size in wave 1 was 17,705. We excluded 13,053 individuals due to presence of MCR at baseline or missing frailty index (FI) data. In the longitudinal analysis, we excluded the participants lacking MCR data in wave 2, which resulted in 3091 eligible individuals. The detailed flowchart of the sample selection process is shown in Figure 1.

### 2.2. Assessment of frailty index

The 2 common clinical approaches to defining frailty are the cumulative deficit index^[20]^ and clinical syndrome.^[21]^ We quantified frailty using the cumulative FI deficit, which reflects the cumulative burden of age-related health deficits. The FI was constructed using the standard deficit accumulation approach, which requires the inclusion of at least 30 health-related deficits.^[22]^ Consistent with established protocols,^[23]^ our FI incorporated 32 deficits across multiple domains: 6 basic activities of daily living, 5 instrumental activities of daily living, sensory impairments (hearing, vision, speech disorders), 14 chronic diseases (including hypertension, dyslipidemia, diabetes, cancer, myocardial infarction, stroke, and kidney disease), 2 disability indicators (physical disability and brain damage), self-reported general health, perceived health changes, 3 mobility limitations, and 5 cognitive deficits assessed using the telephone interview for cognitive status-modified. The telephone interview for cognitive status-modified evaluates cognitive function through assessments of daily memory reports, immediate and delayed recall, calculation tasks, and figure-drawing ability, with total scores ranging from 0 to 21 (higher scores indicate worse cognitive performance). Each deficit item was coded dichotomously (0 = absent, 1 = present). The FI score was calculated as the ratio of present deficits to the total number of items, resulting in a score range of 0 to 1, where higher values indicated greater frailty severity. The FI construction method has been extensively validated in previous studies.^[24,25]^

### 2.3. Outcome ascertainment

MCR was defined as the co-occurrence of subjective cognitive complaints and objectively measured slow gait speed in individuals without dementia or mobility-limiting conditions. Subjective cognitive complaints were assessed using a validated single-item instrument: “How would you describe your current memory status?” Response options included “excellent,” “very good,” “good,” “fair,” and “poor.” Participants endorsing “fair” or “poor” responses were classified as having clinically significant cognitive complaints. Gait speed was assessed using a standardized protocol. Participants completed 2 timed walks along a straight 2.5-m path within a dedicated, unobstructed 4-m walkway to ensure safety and measurement accuracy. Participants were instructed to walk at their usual, comfortable pace, and the use of habitual assistive devices was permitted. The mean completion time across the 2 trials was used to calculate gait speed in meters per second (m/s).

### 2.4. Potential covariates

Based on the established risk factors, we considered the following potential covariates: demographic characteristics, lifestyle factors, and clinical comorbidities. Demographic characteristics included age (years), sex (male/female), marital status (married/unmarried), location (village or city/town), and education level (primary education or below, high school, college, or above). Lifestyle factors included smoking status (current smoker vs non-/ex-smoker), drinking status (never drinking, drinking but less than once a month, drinking more than once a month), and body mass index (BMI; kg/m^2^). Clinical comorbidities (hypertension, diabetes mellitus, stroke, and coronary heart disease) were ascertained based on self-reported medical histories or proxy reports.

### 2.5. Statistical analyses

Descriptive statistics are presented as mean ± standard deviation for continuous variables and as frequencies (%) for categorical variables. Between-group comparisons employed Welch’s *t*-test or analysis of variance for continuous variables and Pearson’s chi-square test or Fisher’s exact test (for expected frequencies <5) for categorical variables. The FI–MCR association was analyzed using multivariable logistic regression, expressed through odds ratios (ORs) and 95% confidence intervals (CIs). We conducted multivariate logistic regression across 3 hierarchical models: model 1 was not adjusted for covariates; model 2 was adjusted for sociodemographic and lifestyle covariates (age, sex, marital status, education, location, smoking, drinking, and BMI); and model 3 was further adjusted for clinical comorbidities (hypertension, diabetes, coronary heart disease, stroke, and depression). Potential nonlinear relationships between FI and MCR risk were examined using restricted cubic splines (RCS) within the logistic regression models. Stratified analyses evaluated effect modification across subgroups defined by: age, sex, marital status, education, location, smoking, drinking, BMI, hypertension, diabetes, coronary heart disease, stroke, and depression. Mediation analysis was performed using the R package “mediation” (version 4.5.0) with 1000 bootstrap iterations to quantify total, direct, and indirect effects (mediated through depressive symptoms) and calculate the mediation proportion. All analyses were performed in R software (version 4.2.2; R Foundation). Statistical significance was defined as 2-tailed *P* < .05.

## 3. Results

### 3.1. Population characteristics

The final cohort comprised 3091 participants aged ≥45 years (Table 1), including 164 with MCR and 2927 without MCR at baseline. The 2-year cumulative incidence of MCR was 5.3%. Significant between-group differences (*P* < .05) were observed between MCR and non-MCR groups in age, marital status, location, stroke, depression, Center for Epidemiological Studies Depression Scale score, and FI (Table 1). Conversely, no statistically significant differences were observed for sex, education, BMI, smoking status, drinking status, hypertension, diabetes mellitus, and coronary heart disease.

**Table 1:** Baseline characteristics of included patients.

Characteristics	Motoric cognitive risk syndrome		*P*-value
	No, N = 2927	Yes, N = 164	
Age, yr (mean ± SD)	66.4 ± 5.9	68.7 ± 6.9	<.001*
Sex			.927†
Male	1510 (51.6%)	84 (51.2%)	
Female	1417 (48.4%)	80 (48.8%)	
Marital			.009†
Married	2427 (82.9%)	123 (75.0%)	
Non-married	500 (17.1%)	41 (25.0%)	
Location			.031†
Village	2352 (80.4%)	143 (87.2%)	
City/town	575 (19.6%)	21 (12.8%)	
Education			.106†
Primary school or below	2415 (82.5%)	145 (88.4%)	
High school	411 (14.0%)	17 (10.4%)	
College or above	101 (3.5%)	2 (1.2%)	
BMI	22.9 ± 3.9	22.5 ± 4.3	.231*
Smoking			.305*
Current smoker	936 (32.0%)	57 (34.8%)	
Non-smoker	1668 (57.0%)	95 (57.9%)	
Ex-smoker	323 (11.0%)	12 (7.3%)	
Drinking			.524†
Drink but less than once a month	186 (6.4%)	9 (5.5%)	
Never drinking	2168 (74.1%)	128 (78.0%)	
drink more than once a month	573 (19.6%)	27 (16.5%)	
Hypertension			.939†
Yes	1294 (44.2%)	72 (43.9%)	
No	1633 (55.8%)	92 (56.1%)	
Diabetes			.657†
Yes	205 (7.0%)	10 (6.1%)	
No	2722 (93.0%)	154 (93.9%)	
Coronary heart disease			.190†
Yes	394 (13.5%)	28 (17.1%)	
No	2533 (86.5%)	136 (82.9%)	
Stroke			.015‡
Yes	65 (2.2%)	9 (5.5%)	
No	2862 (97.8%)	155 (94.5%)	
Depression			.001†
Yes	1106 (37.8%)	83 (50.6%)	
No	1821 (62.2%)	81 (49.4%)	
CES-D score	8 ± 6	11 ± 7	<.001*
Frailty index	0.14 ± 0.10	0.18 ± 0.13	<.001*

BMI = body mass index, CES-D = Center for Epidemiological Studies Depression, SD = standard deviation.

* Welch’s 2-sample *t*-test.

† Pearson’s chi-squared test.

‡ Fisher’s exact test.

### 3.2. Association between FI and MCR

Table 2 presents the association between FI and MCR using a logistic regression. FI was positively associated with MCR in all models: model I (OR = 1.39 [95% CI: 1.21–1.58], *P* < .001); model II (OR = 1.34 [95% CI: 1.17–1.54], *P* < .001); model III (OR = 1.27 [95% CI: 1.07–1.49], *P* < .005). Furthermore, MCR prevalence increased progressively across the higher FI quartiles. Compared with the Q1 (lowest FI) group, the highest quartile (Q4) exhibited significantly elevated odds of MCR (model I: OR = 2.24 [95% CI: 1.44–3.56], *P* < .001, *P* for trend <.001; model II: OR = 2.21 [95% CI: 1.35–3.42], *P* < .001).

**Table 2:** Association between FI and MCR.

Characteristic	Model 1			Model 2			Model 3		
	OR	95% CI	*P*-value	OR	95% CI	*P*-value	OR	95% CI	*P*-value
FI (standardized)	1.39	1.21–1.58	<.001	1.34	1.17–1.54	<.001	1.27	1.07–1.49	.005
FI									
Q1	–	–		–	–		–	–	
Q2	1.10	0.66–1.85	.718	1.14	0.68–1.92	.626	1.08	0.64–1.84	.774
Q3	1.44	0.89–2.35	.141	1.41	0.87–2.31	.172	1.24	0.74–2.11	.421
Q4	2.24	1.44–3.56	<.001	2.12	1.35–3.42	.001	1.71	0.99–2.97	.055
*P* for trend			<.001			<.001			.042

Model 1: No covariates were adjusted.

Model 2: Adjusted for age, sex, marital status, location, education, smoking, drinking, and BMI.

Model 3: Adjusted for age, sex, marital status, location, education, smoking, drinking, BMI, hypertension, diabetes mellitus, stroke, and coronary heart disease, and depression.

BMI = body mass index, CI = confidence interval, FI = frailty index, MCR = motoric cognitive risk, OR = odds ratio.

### 3.3. RCS

**Figure 2.**
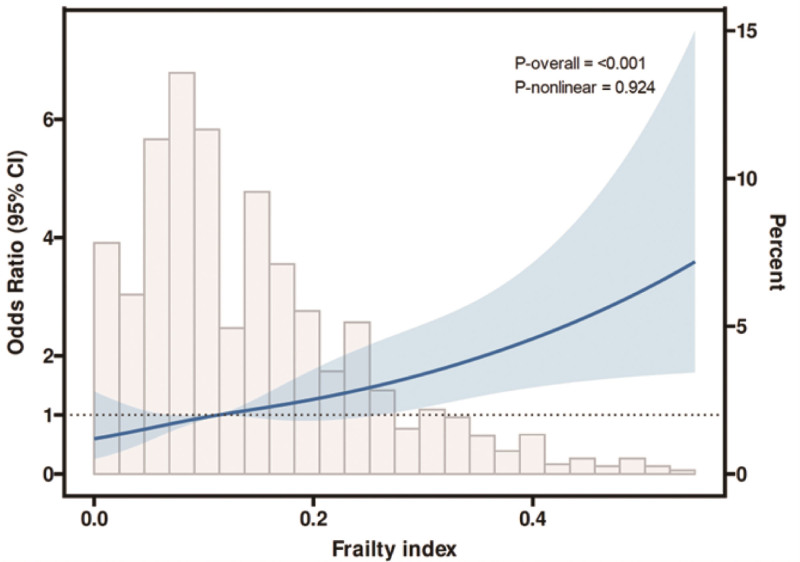
Association between FI and MCR with the RCS function. CI = confidence interval, FI = frailty index, MCR = motoric cognitive risk, RCS = restricted cubic spline.

Figure 2 shows the dose–response relationship of FI with MCR and the prevalence of MCR based on RCS. RCS analysis indicated a linear and positive relationship between FI and MCR after adjusting for all confounding factors (*P* for nonlinearity = .924).

### 3.4. Subgroup analysis

**Figure 3.**
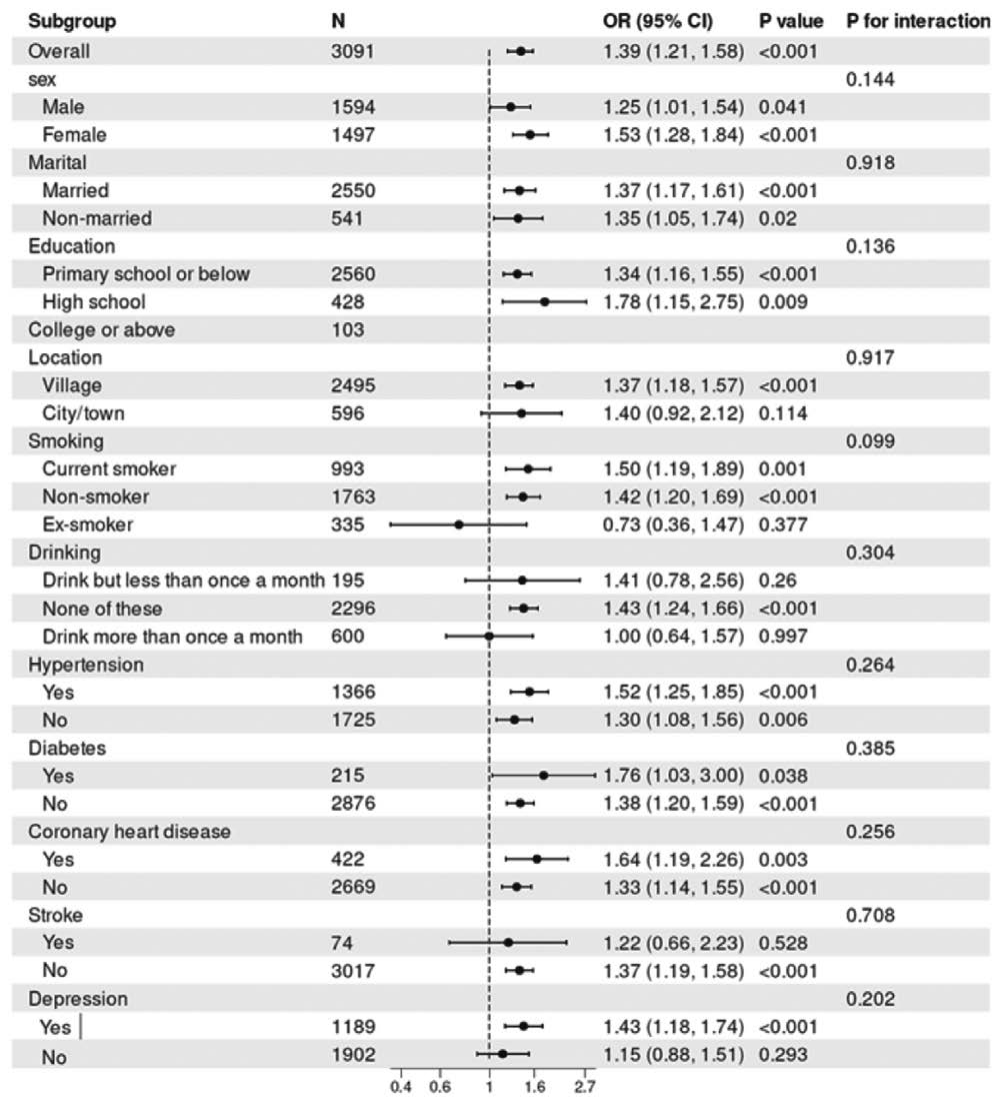
Subgroup analysis of the association between FI and MCR. CI = confidence interval, FI = frailty index, MCR = motoric cognitive risk, OR = odds ratio.

As shown in Figure 3, subgroup analyses assessed the potential effect of modification in the FI–MCR association. No significant interaction effects were observed across age, sex, marital status, location, BMI, education, smoking, drinking, diabetes mellitus, hypertension, coronary heart disease, stroke, or depression (*P* for interaction > .05). These results demonstrated a consistent association between FI and MCR across all population strata.

### 3.5. Mediating effect of depression

Our mediation analysis revealed a statistically significant total effect of frailty on MCR (coefficient = .259; 95% CI: 0.119–0.453; *P* < .001; Table 3). Depressive symptoms significantly mediated the association between FI and MCR prevalence, accounting for 32.1% of the total effect (*P* < .05). After adjusting for depressive symptoms, the direct effect of frailty on MCR remained statistically significant (coefficient = .176; 95% CI: 0.047–0.318; *P* < .001).

**Table 3:** Depressive symptom as a mediator in the associations between FI and MCR.

Independent variable	Mediator	Total effect		Indirect effect		Direct effect		Proportion mediated, % (95% CI)
		Coefficient (95% CI)	*P* value	Coefficient (95% CI)	*P* value	Coefficient (95% CI)	*P* value	
FI	CES-D score	0.25938 (0.11912–0.45261)	<.001	0.08334 (0.00779–0.16284)	<.001	0.17604 (0.04694–0.31783)	<.001	32.1 (5.2–71.5)

The mediation analyses were adjusted for age, sex, marital status, location, education, smoking, drinking, BMI, hypertension, diabetes mellitus, stroke, and coronary heart disease.

BMI = body mass index, CES-D = Center for Epidemiological Studies Depression Scale, CI = confidence interval, FI = frailty index, MCR = motoric cognitive risk.

## 4. Discussion

This study is the first to establish an association between frailty, quantified by a cumulative deficit-based FI, and MCR risk using nationally representative CHARLS data. Our analysis of 3091 CHARLS participants showed significantly higher FI scores in individuals with MCR than in those without MCR. Multivariable logistic regression confirmed a robust positive association, while RCS analysis revealed a linear dose–response relationship. This association remained consistent across all subgroups examined. Crucially, the mediation analysis identified depressive symptoms (quantified by the Center for Epidemiological Studies Depression Scale score) as a significant mediator, suggesting that FI may partially influence MCR risk through depressive pathways. These findings suggest that FI is a promising quantitative biomarker for early MCR detection, potentially enabling targeted preventive intervention.

Although our study is the first to specifically focus on FI–MCR associations in middle-aged and older Chinese adults, prior studies have consistently documented frailty-MCR relationships across diverse populations.^[12-14]^ For example, Shen et al conducted a cross-sectional analysis of 429 Chinese adults (≥60 years) using the clinical frailty scale.^[12]^ After full adjustment, both slow gait (OR 3.40, 95% CI: 1.40–8.23; *P* = .007) and MCR (OR 5.53, 95% CI: 1.46–20.89; *P* = .012) independently predicted frailty.^[12]^ Sathyan et al performed longitudinal assessments in 641 multiethnic older adults (LonGenity study) using a 41-item cumulative deficit FI.^[13]^ Elevated baseline FI predicted incident MCR (hazard ratio: 1.07; 95% CI: 1.03–1.11; *P* = .0002) after covariate adjustment.^[13]^ Similarly, Zhang et al evaluated 3075 adults (age ≥ 65 years) from the National Health and Aging Trends Study using the Makizako Social Frailty Index.^[14]^ Longitudinal data showed that multidomain social frailty increased the incidence of MCR risk (hazard ratio: 1.57, 95% CI: 1.34–1.84) independent of socioeconomic/clinical confounders.^[14]^ Notably, despite methodological heterogeneity (clinical scales vs quantitative indices) and population diversity, these studies collectively demonstrated that frailty consistently potentiates MCR risk.^[12-14]^ This congruence underscores the biological plausibility of health deficit accumulation (as captured by FI) in MCR pathogenesis. Our findings extend this paradigm by establishing FI as a robust, quantifiable biomarker for stratifying the MCR risk in aging populations.

The observed MCR prevalence of 5.3% in our cohort aligns with established epidemiological data (6.4% in prior reports).^[26]^ In line with prior studies,^[12,14]^ our finding indicate that several factors may contribute to MCR, including advanced age, rural residence, spousal absence, stroke history, and depressive symptoms. This risk factor convergence implies shared pathophysiological pathways between frailty and MCR, with depression emerging as a pivotal mechanistic nexus.

Our study proposes a novel pathophysiological framework connecting frailty, depression, and MCR in aging populations, in which frailty precipitates falls, healthcare utilization, social disengagement, and loneliness, culminating in depression, provides a mechanistic foundation.^[27]^ Importantly, our mediation analysis revealed depressive symptoms as a partial mediator in the FI–MCR relationship through 2 distinct neurobehavioral pathways. First, depression adversely affects multiple cognitive domains including executive function, cognitive flexibility, and hippocampal-dependent memory consolidation.^[28]^ Persistent low mood reduces cognitive engagement, while chronic elevation of stress hormones may exert neurotoxic effects on the hippocampal and frontal regions while exacerbating vascular pathology, collectively leading to measurable cognitive decline.^[29,30]^ Supporting this mechanism, a recent meta-analysis indicated that antidepressant therapy for late-life depression improves specific cognitive domains, and this effect is potentially mediated by the alleviation of depressive symptoms.^[31]^ Second, older adults with late-life depression consistently exhibit poorer gait performance than cognitively intact individuals without depression, manifesting as slower gait speed, reduced coordination, and altered gait patterns.^[32]^ These findings suggest that frailty, depressive symptoms, and MCR share overlapping pathophysiological mechanisms and exhibit common susceptibilities to similar stressors, together forming a positive feedback loop that reinforces their interrelationship among these 3 conditions.^[28,33,34]^

While our findings indicate that depressive symptoms partially mediate the frailty-MCR association, the majority of this relationship (67.9%) reflects direct effects, potentially attributable to either frailty itself or other unexamined pathways. A plausible alternative mechanism involves the inflammatory processes. Previous research demonstrates that elevated levels of systemic inflammatory markers, including C-reactive protein and interleukin-6 (IL-6), contribute to sarcopenia and progressive muscle loss, which are key features of frailty.^[35]^ Notably, increased levels of peripheral pro-inflammatory markers (e.g., IL-6, IL-1β, and C-reactive protein) have been consistently associated with a higher MCR prevalence.^[15,36]^ Moreover, these cytokines showed independent associations with both core MCR components: subjective cognitive complaints^[37]^ and objectively measured gait slowing.^[38,39]^ Collectively, these observations suggest that the inflammatory pathways may partially explain the residual direct association between frailty and MCR. Future studies should systematically investigate these pathways and other potential mechanistic pathways underlying this relationship.

Our study has several strengths. First, this is the first investigation to elucidate the relationship between FI and MCR among Chinese middle-aged and older adults using a nationally representative CHARLS database. Second, the population-based prospective cohort design, with its large sample size and long follow-up period, enhanced the reliability and generalizability of our findings to China’s aging population. Third, we comprehensively adjusted for potential confounders and conducted subgroup analyses to ensure robustness of the results. However, several limitations of this study merit consideration. First, excluding participants with incomplete data and using a questionnaire-based frailty assessment could have introduced reporting bias. Second, FI was obtained at a single time point, potentially failing to capture the longitudinal frailty trajectories affecting MCR development. Third, the absence of genetic data in the CHARLS database precluded the assessment of genetic factors affecting frailty-MCR relationships. Further studies that incorporate genetic data are required to validate our findings.

## 5. Conclusion

In conclusion, our findings suggest a positive linear association between FI and MCR in middle-aged and older Chinese adults. Furthermore, our analysis indicates that depressive symptoms may partially mediate this relationship. Future prospective longitudinal studies incorporating biomarkers and neuroimaging data could help clarify potential causal pathways. Further experimental research exploring the mechanistic interactions is required to validate these observations.

## Acknowledgments

We would like to thank all the CHARLS staff for their valuable contributions.

## Author contributions

**Conceptualization:** Yi-Xiao Li, Jing Li.

**Data curation:** Yi-Xiao Li, Jing Li.

**Formal analysis:** Yi-Xiao Li, Jing Li.

**Investigation:** Yi-Xiao Li, Jing Li.

**Methodology:** Yi-Xiao Li.

**Software:** Jing Li.

**Supervision:** Yi-Xiao Li, Jing Li.

**Validation:** Yi-Xiao Li, Jing Li.

**Visualization:** Jing Li.

**Writing – original draft:** Yi-Xiao Li, Jing Li.

**Writing – review & editing:** Yi-Xiao Li.
